# 2-Amino-4-methyl­pyridinium hexa-2,4-dienoate dihydrate

**DOI:** 10.1107/S1600536810033076

**Published:** 2010-08-25

**Authors:** Madhukar Hemamalini, Hoong-Kun Fun

**Affiliations:** a X-ray Crystallography Unit, School of Physics, Universiti Sains Malaysia, 11800 USM, Penang, Malaysia

## Abstract

In the title salt, C_6_H_9_N_2_^+^·C_6_H_7_O_2_^−^·2H_2_O, the non-H atoms of the 2-amino-4-methyl­pyridinium cation are coplanar, with a maximum deviation of 0.010 (1) Å. In the crystal structure, the pyridinium N atom and the 2-amino group of the cation are hydrogen bonded to the carboxyl­ate O atoms of the anion *via* a pair of N—H⋯O hydrogen bonds, forming an *R*_2_^2^(8) ring motif. The sorbate anions and water mol­ecules are linked through O—H⋯O hydrogen bonds, forming *R*_10_^10^(28) and *R*_6_^4^(12) ring motifs. The motifs form part of a three-dimensional framework.

## Related literature

For the role of hydrogen bonding in crystal engineering, see: Goswami & Ghosh (1997 ▶); Goswami *et al.* (1998 ▶); Lehn (1992 ▶). For applications of pyridinium derivatives, see: Akkurt *et al.* (2005 ▶). For details of hydrogen bonding, see: Jeffrey & Saenger (1991 ▶); Jeffrey (1997 ▶); Scheiner (1997 ▶). For hydrogen-bond motifs, see: Bernstein *et al.* (1995 ▶). For bond-length data, see: Allen *et al.* (1987 ▶). For the stability of the temperature controller used in the data collection, see: Cosier & Glazer (1986 ▶).
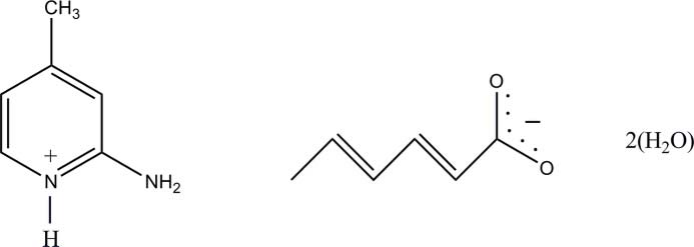


## Experimental

### 

#### Crystal data

C_6_H_9_N_2_^+^·C_6_H_7_O_2_^−^·2H_2_O
*M* *_r_* = 256.30
Monoclinic, 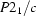

*a* = 8.8233 (4) Å
*b* = 12.6783 (6) Å
*c* = 13.1647 (6) Å
β = 108.279 (1)°
*V* = 1398.35 (11) Å^3^
*Z* = 4
Mo *K*α radiation
μ = 0.09 mm^−1^
*T* = 100 K
0.66 × 0.28 × 0.25 mm

#### Data collection

Bruker APEXII DUO CCD area-detector diffractometer
Absorption correction: multi-scan (*SADABS*; Bruker, 2009 ▶) *T*_min_ = 0.942, *T*_max_ = 0.978
23001 measured reflections
6087 independent reflections
4840 reflections with *I* > 2σ(*I*)
*R*_int_ = 0.031

#### Refinement

*R*[*F* ^2^ > 2σ(*F* ^2^)] = 0.044
*wR*(*F* ^2^) = 0.140
*S* = 1.05
6087 reflections
193 parameters
H atoms treated by a mixture of independent and constrained refinement
Δρ_max_ = 0.41 e Å^−3^
Δρ_min_ = −0.24 e Å^−3^

### 

Data collection: *APEX2* (Bruker, 2009 ▶); cell refinement: *SAINT* (Bruker, 2009 ▶); data reduction: *SAINT*; program(s) used to solve structure: *SHELXTL* (Sheldrick, 2008 ▶); program(s) used to refine structure: *SHELXTL*; molecular graphics: *SHELXTL*; software used to prepare material for publication: *SHELXTL* and *PLATON* (Spek, 2009 ▶).

## Supplementary Material

Crystal structure: contains datablocks global, I. DOI: 10.1107/S1600536810033076/ci5155sup1.cif

Structure factors: contains datablocks I. DOI: 10.1107/S1600536810033076/ci5155Isup2.hkl

Additional supplementary materials:  crystallographic information; 3D view; checkCIF report

## Figures and Tables

**Table 1:** Hydrogen-bond geometry (Å, °)

*D*—H⋯*A*	*D*—H	H⋯*A*	*D*⋯*A*	*D*—H⋯*A*
N1—H1*N*1⋯O2^i^	0.97 (2)	1.72 (2)	2.6875 (9)	175 (1)
N2—H1*N*2⋯O1^i^	0.91 (2)	2.01 (2)	2.9139 (10)	173 (1)
N2—H2*N*2⋯O1*W*	0.94 (2)	1.92 (2)	2.8453 (11)	166 (1)
O2*W*—H1*W*2⋯O2^ii^	0.85 (2)	1.91 (2)	2.7510 (10)	167 (2)
O2*W*—H2*W*2⋯O1	0.87 (2)	1.96 (2)	2.8140 (9)	168 (2)
O1*W*—H1*W*1⋯O1^iii^	0.84 (2)	2.05 (2)	2.8777 (10)	168 (2)
O1*W*—H2*W*1⋯O2*W*	0.86 (2)	1.88 (2)	2.7425 (11)	173 (2)

Symmetry codes: (i) 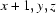; (ii) 
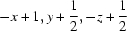
; (iii) 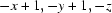.

**Fig. 1.**
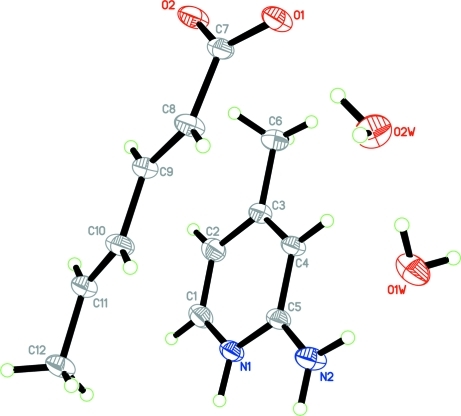
The asymmetric unit of the title compound. Displacement ellipsoids are drawn at the 50% probability level.

**Fig. 2.**
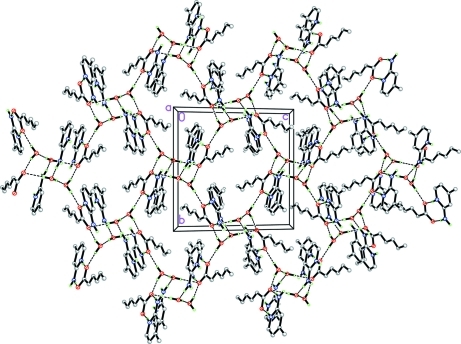
The crystal packing of the title compound, showing part of a hydrogen-bonded (dashed lines) three-dimensional network. H atoms not involved in the interactions have been omitted for clarity.

**Fig. 3.**
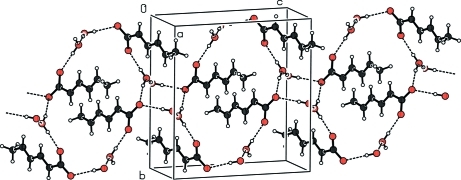
Part of a hydrogen-bonded (dashed lines) two-dimensional network made up of anions and water molecules.

## supplementary crystallographic information

### Comment

Hydrogen bonding plays a key role in molecular recognition (Goswami & Ghosh, 1997) and crystal engineering research (Goswami *et al.*, 1998). The design of highly specific solid-state compounds is of considerable significance in organic chemistry due to important applications of these compounds in the development of new optical, magnetic and electronic systems (Lehn, 1992). Pyridinium derivatives often possess antibacterial and antifungal activities (Akkurt *et al.*, 2005). They are often involved in hydrogen-bonding interactions (Jeffrey & Saenger, 1991; Jeffrey, 1997; Scheiner, 1997). In order to study some hydrogen bonding interactions, the synthesis and structure of the title salt, (I), is presented here.

The asymmetric unit of (I) contains one 2-amino-4-methylpyridinium cation, one sorbate anion and two water molecules (Fig. 1). The non-H atoms of the 2-amino-4-methylpyridinium cation are coplanar, with a maximum deviation of 0.010 (1) Å for atom N1. The protonation of atom N1 has lead to a slight increase in the C1—N1—C5 angle to 121.96 (6)°. The bond lengths (Allen *et al.*, 1987) and angles are within normal ranges.

In the crystal packing (Fig. 2), the protonated N1 atom and one of the 2-amino group hydrogen (H1N2) are hydrogen-bonded to the carboxylate oxygen atoms (O1 and O2) via a pair of intermolecular N1—H1N1···O2 and N2—H1N2···O1 hydrogen bonds forming an *R*_2_^2^(8) ring motif (Bernstein *et al.*, 1995). The sorbate anion and two water molecules are linked through O2W—H1W2···O2, O2W—H2W2···O1, O1W—H1W1···O1 and O1W—H2W1···O2W (Table 1) hydrogen-bonds, forming *R*_10_^10^(28) and *R*_6_^4^(12) ring motifs (Fig. 3).

### Experimental

A hot methanol solution (20 ml) of 2-amino-4-methylpyridine (54 mg, Aldrich) and sorbic acid (56 mg, Merck) were mixed and warmed over a heating magnetic stirrer hotplate for a few minutes. The resulting solution was allowed to cool slowly at room temperature and crystals of the title compound appeared after a few days.

### Refinement

Atoms H1N1, H1N2, H2N2, H1W2, H2W2, H1W1 and H2W1 were located in a difference Fourier map and were refined freely [N–H= 0.911 (18)– 0.967 (17) Å and O–H= 0.84 (18)–0.87 (2) Å]. The remaining H atoms were positioned geometrically [C–H = 0.93 or 0.96 Å] and were refined using a riding model, with *Uiso*(H) = 1.2 or 1.5 *Ueq*(C). A rotating group model was used for the methyl group.

### Crystal data

**Table:**

C_6_H_9_N_2_^+^·C_6_H_7_O_2_^−^·2H_2_O	*F*(000) = 552
*M**_r_* = 256.30	*D*_x_ = 1.217 Mg m^−^^3^
Monoclinic, *P*2_1_/*c*	Mo *K*α radiation, λ = 0.71073 Å
Hall symbol: -P 2ybc	Cell parameters from 7645 reflections
*a* = 8.8233 (4) Å	θ = 2.9–34.9°
*b* = 12.6783 (6) Å	µ = 0.09 mm^−^^1^
*c* = 13.1647 (6) Å	*T* = 100 K
β = 108.279 (1)°	Needle, brown
*V* = 1398.35 (11) Å^3^	0.66 × 0.28 × 0.25 mm
*Z* = 4	

### Data collection

**Table:**

Bruker APEXII DUO CCD area-detector diffractometer	6087 independent reflections
Radiation source: fine-focus sealed tube	4840 reflections with *I* > 2σ(*I*)
graphite	*R*_int_ = 0.031
φ and ω scans	θ_max_ = 35.1°, θ_min_ = 2.9°
Absorption correction: multi-scan (*SADABS*; Bruker, 2009)	*h* = −14→14
*T*_min_ = 0.942, *T*_max_ = 0.978	*k* = −20→20
23001 measured reflections	*l* = −21→21

### Refinement

**Table:**

Refinement on *F*^2^	Primary atom site location: structure-invariant direct methods
Least-squares matrix: full	Secondary atom site location: difference Fourier map
*R*[*F*^2^ > 2σ(*F*^2^)] = 0.044	Hydrogen site location: inferred from neighbouring sites
*wR*(*F*^2^) = 0.140	H atoms treated by a mixture of independent and constrained refinement
*S* = 1.05	*w* = 1/[σ^2^(*F*_o_^2^) + (0.0809*P*)^2^ + 0.1516*P*] where *P* = (*F*_o_^2^ + 2*F*_c_^2^)/3
6087 reflections	(Δ/σ)_max_ = 0.001
193 parameters	Δρ_max_ = 0.41 e Å^−^^3^
0 restraints	Δρ_min_ = −0.24 e Å^−^^3^

### Special details

**Table:**

Experimental. The crystal was placed in the cold stream of an Oxford Cryosystems Cobra open-flow nitrogen cryostat (Cosier & Glazer, 1986) operating at 100.0 (1) K.
Geometry. All esds (except the esd in the dihedral angle between two l.s. planes) are estimated using the full covariance matrix. The cell esds are taken into account individually in the estimation of esds in distances, angles and torsion angles; correlations between esds in cell parameters are only used when they are defined by crystal symmetry. An approximate (isotropic) treatment of cell esds is used for estimating esds involving l.s. planes.
Refinement. Refinement of F^2^ against ALL reflections. The weighted R-factor wR and goodness of fit S are based on F^2^, conventional R-factors R are based on F, with F set to zero for negative F^2^. The threshold expression of F^2^ > 2sigma(F^2^) is used only for calculating R-factors(gt) etc. and is not relevant to the choice of reflections for refinement. R-factors based on F^2^ are statistically about twice as large as those based on F, and R- factors based on ALL data will be even larger.

### Fractional atomic coordinates and isotropic or equivalent isotropic displacement parameters (Å^2^)

**Table:**

	*x*	*y*	*z*	*U*_iso_*/*U*_eq_	
N1	1.07997 (7)	0.26852 (5)	0.19290 (5)	0.02127 (12)	
N2	1.01177 (8)	0.43659 (6)	0.12630 (6)	0.02585 (14)	
C1	1.04257 (9)	0.16859 (7)	0.21363 (7)	0.02539 (15)	
H1A	1.1237	0.1224	0.2494	0.030*	
C2	0.88836 (10)	0.13495 (7)	0.18292 (7)	0.02690 (16)	
H2A	0.8638	0.0664	0.1974	0.032*	
C3	0.76591 (9)	0.20597 (7)	0.12849 (6)	0.02333 (14)	
C4	0.80544 (8)	0.30662 (6)	0.10894 (6)	0.02151 (14)	
H4A	0.7257	0.3538	0.0734	0.026*	
C5	0.96682 (8)	0.33929 (6)	0.14240 (6)	0.01984 (13)	
C6	0.59508 (10)	0.17064 (8)	0.09388 (8)	0.03179 (18)	
H6A	0.5284	0.2258	0.0535	0.048*	
H6B	0.5638	0.1549	0.1558	0.048*	
H6C	0.5836	0.1087	0.0502	0.048*	
O1	0.35573 (6)	0.47026 (5)	0.19351 (5)	0.02451 (12)	
O2	0.39446 (7)	0.30966 (5)	0.26595 (6)	0.02668 (13)	
C7	0.44389 (8)	0.40024 (6)	0.25192 (6)	0.02007 (13)	
C8	0.61399 (8)	0.42857 (6)	0.30428 (7)	0.02356 (15)	
H8A	0.6422	0.4993	0.3054	0.028*	
C9	0.72932 (8)	0.35883 (6)	0.34995 (6)	0.02082 (13)	
H9A	0.7010	0.2886	0.3538	0.025*	
C10	0.89651 (8)	0.38771 (7)	0.39373 (6)	0.02347 (14)	
H10A	0.9226	0.4589	0.3960	0.028*	
C11	1.01497 (9)	0.31857 (7)	0.43086 (6)	0.02396 (15)	
H11A	0.9882	0.2476	0.4307	0.029*	
C12	1.18754 (9)	0.34726 (8)	0.47266 (7)	0.02996 (18)	
H12A	1.2317	0.3219	0.5446	0.045*	
H12B	1.1986	0.4226	0.4719	0.045*	
H12C	1.2434	0.3158	0.4284	0.045*	
O1W	0.75170 (8)	0.57495 (6)	0.03208 (6)	0.03181 (15)	
O2W	0.51394 (8)	0.62847 (5)	0.11569 (6)	0.03052 (14)	
H1N1	1.192 (2)	0.2876 (12)	0.2177 (14)	0.047 (4)*	
H1N2	1.118 (2)	0.4529 (13)	0.1473 (14)	0.049 (4)*	
H2N2	0.9370 (19)	0.4873 (12)	0.0898 (13)	0.042 (4)*	
H1W2	0.529 (2)	0.6833 (14)	0.1552 (15)	0.051 (4)*	
H2W2	0.468 (2)	0.5855 (14)	0.1483 (13)	0.049 (4)*	
H1W1	0.7068 (19)	0.5623 (12)	−0.0331 (14)	0.041 (4)*	
H2W1	0.672 (2)	0.5876 (14)	0.0553 (16)	0.061 (5)*	

### Atomic displacement parameters (Å^2^)

**Table:**

	*U*^11^	*U*^22^	*U*^33^	*U*^12^	*U*^13^	*U*^23^
N1	0.0137 (2)	0.0268 (3)	0.0213 (3)	0.0011 (2)	0.00269 (19)	−0.0009 (2)
N2	0.0161 (3)	0.0250 (3)	0.0347 (4)	0.0000 (2)	0.0054 (2)	0.0003 (3)
C1	0.0194 (3)	0.0278 (3)	0.0252 (3)	0.0014 (2)	0.0017 (3)	0.0030 (3)
C2	0.0219 (3)	0.0289 (4)	0.0267 (3)	−0.0031 (3)	0.0031 (3)	0.0042 (3)
C3	0.0158 (3)	0.0327 (4)	0.0203 (3)	−0.0031 (2)	0.0038 (2)	0.0002 (3)
C4	0.0125 (3)	0.0294 (3)	0.0216 (3)	0.0009 (2)	0.0038 (2)	−0.0002 (2)
C5	0.0139 (3)	0.0252 (3)	0.0199 (3)	0.0011 (2)	0.0046 (2)	−0.0023 (2)
C6	0.0178 (3)	0.0439 (5)	0.0312 (4)	−0.0086 (3)	0.0041 (3)	0.0033 (3)
O1	0.0151 (2)	0.0244 (3)	0.0303 (3)	0.00155 (18)	0.00176 (19)	0.0031 (2)
O2	0.0139 (2)	0.0262 (3)	0.0363 (3)	−0.00069 (18)	0.0026 (2)	0.0061 (2)
C7	0.0130 (2)	0.0238 (3)	0.0224 (3)	0.0010 (2)	0.0039 (2)	−0.0004 (2)
C8	0.0134 (3)	0.0252 (3)	0.0292 (3)	−0.0009 (2)	0.0027 (2)	0.0005 (3)
C9	0.0137 (3)	0.0264 (3)	0.0218 (3)	0.0000 (2)	0.0047 (2)	0.0005 (2)
C10	0.0134 (3)	0.0282 (3)	0.0268 (3)	−0.0005 (2)	0.0034 (2)	0.0012 (3)
C11	0.0143 (3)	0.0332 (4)	0.0230 (3)	0.0010 (2)	0.0038 (2)	0.0006 (3)
C12	0.0128 (3)	0.0450 (5)	0.0293 (4)	0.0016 (3)	0.0026 (3)	0.0013 (3)
O1W	0.0235 (3)	0.0377 (3)	0.0302 (3)	0.0067 (2)	0.0027 (2)	−0.0046 (3)
O2W	0.0328 (3)	0.0252 (3)	0.0357 (3)	−0.0037 (2)	0.0138 (3)	−0.0036 (2)

### Geometric parameters (Å, °)

**Table:**

N1—C5	1.3518 (9)	O2—C7	1.2624 (9)
N1—C1	1.3583 (11)	C7—C8	1.4859 (10)
N1—H1N1	0.967 (17)	C8—C9	1.3397 (10)
N2—C5	1.3329 (10)	C8—H8A	0.93
N2—H1N2	0.911 (18)	C9—C10	1.4522 (10)
N2—H2N2	0.938 (16)	C9—H9A	0.93
C1—C2	1.3607 (11)	C10—C11	1.3339 (11)
C1—H1A	0.93	C10—H10A	0.93
C2—C3	1.4163 (12)	C11—C12	1.4927 (11)
C2—H2A	0.93	C11—H11A	0.93
C3—C4	1.3684 (11)	C12—H12A	0.96
C3—C6	1.4997 (11)	C12—H12B	0.96
C4—C5	1.4141 (10)	C12—H12C	0.96
C4—H4A	0.93	O1W—H1W1	0.840 (18)
C6—H6A	0.96	O1W—H2W1	0.87 (2)
C6—H6B	0.96	O2W—H1W2	0.853 (18)
C6—H6C	0.96	O2W—H2W2	0.868 (17)
O1—C7	1.2686 (9)		
			
C5—N1—C1	121.96 (6)	H6A—C6—H6C	109.5
C5—N1—H1N1	121.1 (9)	H6B—C6—H6C	109.5
C1—N1—H1N1	116.9 (9)	O2—C7—O1	123.44 (6)
C5—N2—H1N2	119.3 (11)	O2—C7—C8	119.81 (6)
C5—N2—H2N2	121.3 (9)	O1—C7—C8	116.75 (7)
H1N2—N2—H2N2	119.3 (14)	C9—C8—C7	124.27 (7)
N1—C1—C2	121.07 (7)	C9—C8—H8A	117.9
N1—C1—H1A	119.5	C7—C8—H8A	117.9
C2—C1—H1A	119.5	C8—C9—C10	123.08 (7)
C1—C2—C3	118.95 (8)	C8—C9—H9A	118.5
C1—C2—H2A	120.5	C10—C9—H9A	118.5
C3—C2—H2A	120.5	C11—C10—C9	124.16 (8)
C4—C3—C2	119.28 (7)	C11—C10—H10A	117.9
C4—C3—C6	120.83 (7)	C9—C10—H10A	117.9
C2—C3—C6	119.89 (8)	C10—C11—C12	124.50 (8)
C3—C4—C5	120.38 (7)	C10—C11—H11A	117.7
C3—C4—H4A	119.8	C12—C11—H11A	117.7
C5—C4—H4A	119.8	C11—C12—H12A	109.5
N2—C5—N1	118.80 (6)	C11—C12—H12B	109.5
N2—C5—C4	122.84 (7)	H12A—C12—H12B	109.5
N1—C5—C4	118.35 (7)	C11—C12—H12C	109.5
C3—C6—H6A	109.5	H12A—C12—H12C	109.5
C3—C6—H6B	109.5	H12B—C12—H12C	109.5
H6A—C6—H6B	109.5	H1W1—O1W—H2W1	102.7 (17)
C3—C6—H6C	109.5	H1W2—O2W—H2W2	102.4 (15)
			
C5—N1—C1—C2	0.83 (12)	C3—C4—C5—N2	−179.36 (8)
N1—C1—C2—C3	0.02 (13)	C3—C4—C5—N1	0.62 (11)
C1—C2—C3—C4	−0.51 (13)	O2—C7—C8—C9	13.47 (12)
C1—C2—C3—C6	180.00 (8)	O1—C7—C8—C9	−165.81 (8)
C2—C3—C4—C5	0.19 (12)	C7—C8—C9—C10	175.41 (7)
C6—C3—C4—C5	179.67 (7)	C8—C9—C10—C11	−173.75 (8)
C1—N1—C5—N2	178.84 (8)	C9—C10—C11—C12	177.89 (8)
C1—N1—C5—C4	−1.14 (11)		

### Hydrogen-bond geometry (Å, °)

**Table:**

*D*—H···*A*	*D*—H	H···*A*	*D*···*A*	*D*—H···*A*
N1—H1N1···O2^i^	0.97 (2)	1.72 (2)	2.6875 (9)	175 (1)
N2—H1N2···O1^i^	0.91 (2)	2.01 (2)	2.9139 (10)	173 (1)
N2—H2N2···O1W	0.94 (2)	1.92 (2)	2.8453 (11)	166 (1)
O2W—H1W2···O2^ii^	0.85 (2)	1.91 (2)	2.7510 (10)	167 (2)
O2W—H2W2···O1	0.87 (2)	1.96 (2)	2.8140 (9)	168 (2)
O1W—H1W1···O1^iii^	0.84 (2)	2.05 (2)	2.8777 (10)	168 (2)
O1W—H2W1···O2W	0.86 (2)	1.88 (2)	2.7425 (11)	173 (2)

Symmetry codes: (i) *x*+1, *y*, *z*; (ii) −*x*+1, *y*+1/2, −*z*+1/2; (iii) −*x*+1, −*y*+1, −*z*.

## References

[bb1] Akkurt, M., Karaca, S., Jarrahpour, A. A., Zarei, M. & Büyükgüngör, O. (2005). *Acta Cryst.* E**61**, o776–o778.

[bb2] Allen, F. H., Kennard, O., Watson, D. G., Brammer, L., Orpen, A. G. & Taylor, R. (1987). *J. Chem. Soc. Perkin Trans. 2*, pp. S1–19.

[bb3] Bernstein, J., Davis, R. E., Shimoni, L. & Chang, N.-L. (1995). *Angew. Chem. Int. Ed. Engl.* **34**, 1555–1573.

[bb4] Bruker (2009). *APEX2*, *SAINT* and *SADABS* Bruker AXS Inc., Madison, Wisconsin, USA.

[bb5] Cosier, J. & Glazer, A. M. (1986). *J. Appl. Cryst.* **19**, 105–107.

[bb6] Goswami, S. P. & Ghosh, K. (1997). *Tetrahedron Lett.* **38**, 4503–4506.

[bb7] Goswami, S., Mahapatra, A. K., Nigam, G. D., Chinnakali, K. & Fun, H.-K. (1998). *Acta Cryst.* C**54**, 1301–1302.

[bb8] Jeffrey, G. A. (1997). *An Introduction to Hydrogen Bonding.* Oxford University Press.

[bb9] Jeffrey, G. A. & Saenger, W. (1991). *Hydrogen Bonding in Biological Structures.* Berlin: Springer.

[bb10] Lehn, J. M. (1992). *J. Coord. Chem.* **27**, 3–6.

[bb11] Scheiner, S. (1997). *Hydrogen Bonding. A Theoretical Perspective.* Oxford University Press.

[bb12] Sheldrick, G. M. (2008). *Acta Cryst.* A**64**, 112–122. 10.1107/S0108767307043930 18156677

[bb13] Spek, A. L. (2009). *Acta Cryst.* D**65**, 148–155. 10.1107/S090744490804362X PMC2631630 19171970

